# Distinct dynamics of the nucleolus in response to nutrient availability and during development in the rice blast fungus

**DOI:** 10.1128/mbio.01844-23

**Published:** 2023-09-28

**Authors:** Eunbyeol Cho, Song Hee Lee, Ralph A. Dean, Junhyun Jeon

**Affiliations:** 1 Department of Biotechnology, College of Life and Applied Sciences, Yeungnam University, Gyeongsan, Gyeongbuk, South Korea; 2 Plant Immunity Research Center, Seoul National University, Seoul, South Korea; 3 Department of Entomology and Plant Pathology, North Carolina State University, Raleigh, North Carolina, USA; Universidad de Cordoba, Cordoba, Spain

**Keywords:** nucleolus, filamentous fungi, nutrient availability, fungal development, rice blast

## Abstract

The nucleolus has emerged as a central hub for nuclear functions in eukaryotes. Studies in mammals and the model yeast *Saccharomyces cerevisiae* showed that the nucleolus is pivotal to nuclear functions other than ribosome biogenesis. Importantly, rDNA transcription and ribosome assembly were shown to positively correlate with the size of the nucleolus. However, little is known about the nucleolus of filamentous fungi. Here, we investigated how the size and shape of nucleolus in the plant pathogenic filamentous fungus, *Magnaporthe oryzae,* respond to nutrient conditions and infection-related development by monitoring the nucleolar marker protein, MoNOP1 tagged with red fluorescent protein (MoNOP1-RFP). Our work revealed that in the hyphae of *M. oryzae,* the nucleolar size is decoupled from the nucleolar activity under low nutrient condition. A complete lack of carbon or nitrogen did not cause such decoupling. In conidia, no or faint RFP signals were initially observed, hinting at the absence of functional nucleoli. RFP signals only started to become visible in conidia within 2 hours of germination when the germ tube tip began to differentiate a specialized infection structure, the appressorium. RFP signals diminished thereafter. In contrast, in the appressorium, signals became concentrated after 6 hours as the appressorium matured. We propose that such nucleolar dynamics may reflect the strategy of filamentous fungi under low nutrient availability to forage for food resources as well as the roles of the nucleolus during fungal development.

**IMPORTANCE:**

The nucleolus is a dynamic subnuclear structure that is involved in many fundamental processes of the nucleus. In higher eukaryotic cells, the size and shape of nucleoli correlate with nucleolar activities. For fungi, knowledge of the nucleolus and its functions is primarily gleaned from budding yeast. Whether such correlation is conserved and how nucleolar functions are regulated in filamentous fungi including important human and crop pathogens are largely unknown. Our observations reveal that the dynamics of nucleolus in a model plant pathogenic fungus, *Magnaporthe oryzae,* is distinct from those of animal and yeast nucleoli under low nutrient availability and during pathogenic development. Our data not only provide new insight into the nucleoli in filamentous fungi but also highlight the need for investigating how nucleolar dynamics is regulated in comparison to other eukaryotes.

## OBSERVATION

The nucleolus is a most conspicuous subnuclear body that forms by a phenomenon called liquid-liquid phase separation and thus is not bound by a membrane (1 – 3). It is best known for its role in ribosome biogenesis (4 – 6). During the last three decades, however, it has been shown that the nucleolus is a multifunctional organelle involved in, but not limited to, signal recognition particle assembly, small RNA modifications, sensing of cellular stresses, sequestration of proteins, and DNA damage response (7, 8). This clearly points to the nucleolus as a central organizing hub for many nuclear functions (2).

The nucleolus exhibits dynamic changes in both its size and morphology (9, 10). Assembly and disassembly of the nucleolus are coordinated in close connection to the cell cycle progression through the activation and inactivation of RNA Pol I transcriptional machinery, which is under the control of cell cycle checkpoint components such as cyclin-dependent kinases (CDKs) and PP1 phosphatases (11). The size of the nucleolus is influenced by nutrient availability through the target of rapamycin (TOR) signaling pathway, making it an indirect indicator of ribosome biogenesis activity within the nucleolus (12 – 14). Low nutrient availability is known to cause reduction in the size of nucleolus in both yeast and animal cells (14). Notably, metabolically active cells and most cancer cells tend to have enlarged and increased number of nucleoli (15, 16). However, little is known about the nucleoli of filamentous fungi to date, despite their evolutionary and functional implications. In this study, we used a nucleolar marker protein MoNOP1 (17, 18) to monitor the changes in the shape and size of nucleoli during nutrient deprivation and pathogenic development in a model plant pathogenic filamentous fungus, *Magnaporthe oryzae*. The fungus is a causal agent of the rice blast, the most devastating disease in cultivated rice (19).

**Fig 1:**
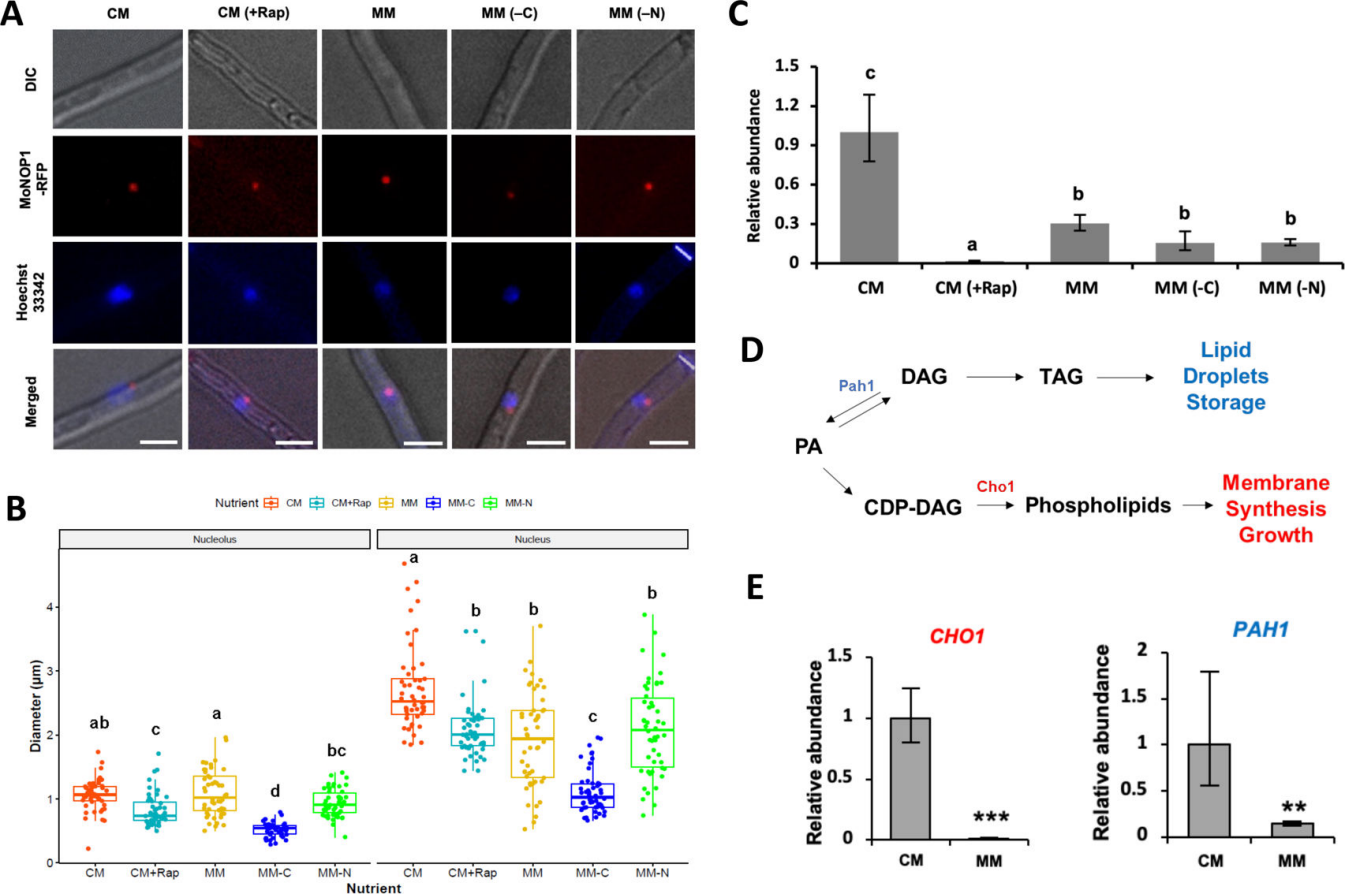
Localization and size of MoNOP1-RFP in response to nutrient availability and rapamycin treatment. (**A**) MoNOP1-RFP signals from the hyphae grown in complete medium (CM), CM supplemented with rapamycin (CM (+Rap)), minimal medium (MM), MM without carbon source (MM (−C)), and MM without nitrogen source (MM (−N)). Fungal nuclei were visualized by staining with Hoechst 33342. Hyphae of strains expressing the MoNOP1-RFP constructs were examined under differential interference contrast (DIC) and fluorescence microscope. Scale bar = 10 µm. (**B**) Sizes of nucleoli and nuclei estimated by measuring the diameter of the MoNOP1 and Hoechst 33342 signals. (**C**) Relative abundances of rRNA in different nutrient conditions measured by qRT-PCR. Different letters indicate statistically significant differences in mean values (*P* < 0.05, ANOVA followed by Tukey’s test). (**D**) Schematic diagram of genetic pathway for nuclear membrane metabolism involving *PAH1* and *CHO1*. (**E**) Relative transcript abundance of *CHO1* and *PAH1* measured by qRT-PCR under different nutrient availability. Error bars indicate standard errors.

The NOP1 in yeast is a snoRNP (small nucleolar ribonucleoprotein) that is involved in the modification of pre-rRNA (18). Therefore, NOP1 is localized to the nucleolus, and it closely correlates with the degree of rRNA synthesis activity of the nucleolus. In the previous work, *MoNOP1* (*M. oryzae NOP1*) had been used to show the exclusion of the RNA-binding protein, RBP35 from the nucleolus (17). In our approach, we generated MoNOP1-RFP (red fluorescent protein) translational fusion, of which the expression is driven by the native promoter of *MoNOP1* gene, and confirmed that our strain expressing *MoNOP1-RFP* has no defects in vegetative growth, asexual reproduction, pathogenic development, and virulence (Fig. S1A through G). Western blot analysis showed that MoNOP1-RFP proteins are intact and stable in complete (CM) and minimal media (MM) condition (Fig. S1H). The RFP signal in hyphae grown in the nutrient-rich media (CM) was visible as a small red dot within the fungal nucleus as previously shown, indicating that MoNOP1-RFP marks the location of the nucleolus (Fig. 1A, left panels).

However, the nucleoli in hyphae grown in the nutrient poor media (MM) appeared to be similar to or even larger than those in CM (CM and MM columns in Fig. 1A). This seemed to contradict the previous observations of nucleolar size changes in response to the nutrient availability in the budding yeast and animal cells. To get more insight into this phenomenon, we monitored and measured the diameter of both nucleoli and nuclei under more specific conditions. When we first tested the effect of decreasing concentrations of nutrients by using CM, MM, and 1/2 MM (half concentration of MM), our quantitative analysis revealed that the size of nucleoli did not change significantly with varying nutrient concentration (Fig. S2A). To our surprise, it was the size of nuclei that decreased as nutrients became scarce, making the relative size of nucleoli appear to be larger under a microscope than they actually were. It should be noted that the RFP signals were not clearly visible in the hyphal tips, where mitosis actively takes place. When we induced S-phase cell cycle arrest using hydroxyurea and observed the RFP signal again in hyphae grown in CM and MM, we were able to see the identical pattern of signals at the hyphal tips as well (Fig. S2B and C). This indicated that disassembly of nucleoli during mitosis is responsible for the near absence of RFP signals at the hyphal tips and that changes in nucleus size in response to the nutrient availability is not dependent on the cell cycle.

In addition to the nutrient concentrations, we tested the effect of rapamycin treatment and lack of either carbon or nitrogen in the media (CM (+Rap), MM (−C), and MM (−N) columns in Fig. 1A). Rapamycin was able to decrease the sizes of both nucleoli and nuclei, indicating the conserved role of the TOR kinase in the regulation of nucleolus (Fig. 1B). Interestingly, nutrient deficiencies seem to affect the size of nucleoli and nuclei as well. Absence of carbon in the media, in particular, had the biggest impact. These results suggest that *M. oryzae* maintains the size of nucleolus under low nutrient availability, unless its environment completely lacks either carbon or nitrogen.

As the size of the nucleolus is known to correlate with rRNA transcription activity, we examined the steady-state levels of rRNA in hyphae grown in different nutrient conditions. The measurement of rRNA transcript abundance showed that rapamycin treatment and nutrient deficiency lead to reduced rRNA abundances. This is expected, as the size of nucleolus decreases under those conditions. However, we found that under low nutrient concentration (MM) in which the size of nucleolus remained unchanged, rRNA abundance decreases (Fig. 1C). This may suggest that regulations of nucleolar size and activity are decoupled under low nutrient availability.

We reasoned that if a decrease of nuclear size in MM is true, then we should be able to see concomitant changes in nuclear envelope (NE) synthesis. In *S. cerevisiae*, the biosynthesis of phospholipids is regulated in large part by the two key enzymes, CHO1 and PAH1, which are involved in membrane biogenesis and synthesis of storage lipids, respectively (20, 21). Based on this, we predicted that the expression of both genes should be downregulated in MM, compared to CM. To check whether the observed reduction in nuclear size is in accordance with decreased phospholipid biosynthesis and storage under low nutrient availability, we identified the orthologs of two proteins in *M. oryzae* and measured the relative abundance of transcript levels of *MoCHO1* (MGG_03550) and *MoPAH1* (MGG_01311). Our qRT-PCR analysis showed that the transcript abundance of *MoCHO1* and *MoPAH1* was significantly lower in MM than that in CM (Fig. 1D and E), supporting our observation at the molecular level.

**Fig 2:**
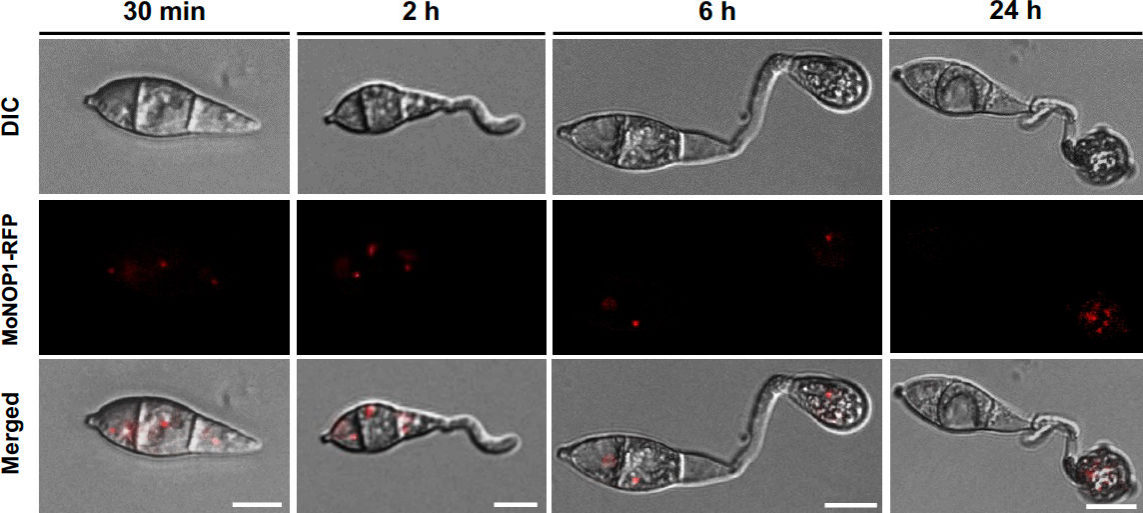
Localization and size of MoNOP1-RFP signals during infection-related development in *Magnaporthe oryzae*. Conidia of strains expressing the MoNOP1-RFP constructs were examined under differential interference contrast (DIC) and fluorescence microscopy. The bottom image was generated by merging the DIC and fluorescence images. During development, fluorescence signals were monitored at 0.5, 2, 6, and 24 hours post-incubation. Scale bar = 10 µm.

Next, we monitored the dynamics of the nucleolus during pathogenic development. A conidium, an asexual spore of *M. oryzae,* initiates germination, after tightly adhering to the substratum (19, 22). The germ tube then ceases to grow, following the recognition of surface hardness and hydrophobicity, and starts to differentiate an infection-specific structure, called an appressorium, via isotropic swelling of its tip (23, 24). The appressorium is a dome-shaped, single-celled structure that can generate and channel chemical pressure into mechanical pressure in order to infect the host plants by breaching their cuticular layer (25, 26). In conidia on an inductive surface (plastic coverslip, hydrophobic), MoNOP1-RFP signal was hardly visible until around 2 hours post-incubation (hpi) (Fig. 2). During the early stage of appressorium formation (4–6 hpi), the RFP signals in conidia became diffused, while in appressoria, most of the appressoria showed signals in the cell periphery and what appears to be nucleolus, with less than 10% of appressoria displaying signals in speckled form (Fig. S3A and B). However, such ratio was reversed when appressoria were observed at 24 hpi. More than 90% of appressoria showed RFP signals in speckled form later on during the development. It is not clear what these speckles are, as there have been no reports on such structures to date and the speckles don’t seem to be confined within nucleus. Our staining of vacuoles with CMAC (7-amino-4-chloromethylcoumarin) showed that the speckles display distinct pattern from distribution of vacuoles, suggesting that the speckles are unlikely to result from the degradation of MoNOP1-RFP in vacuoles (Fig. S3C). Later (6–8 hpi and onward), the RFP signals in conidia gradually disappeared as the conidia underwent autophagic cell death (27), and the signals in appressoria tended to aggregate into one that appeared to be a nucleolus.

A previous study based on the use of transcription and translation inhibitors reported that the initiation and elongation of the germ tube do not require new transcription but new translation of mRNAs in *M. oryzae* (28). We confirmed in our own experiment using transcription and translation inhibitors that germination of conidia requires *de novo* translation but not transcription in *M. oryzae* (Fig. S4). A near absence of MoNOP1-RFP signal in the nascent and germinating conidia may imply that not just mRNAs but also rRNAs are ready-made and loaded into the developing conidia during conidiogenesis and that conidial germination is not dependent on the *de novo* transcription of rDNA. The pattern of RFP signals during the formation of mature appressorium suggests that appressorium formation correlates with nucleolar activity, which is supported by our observation that the addition of a translation inhibitor, cycloheximide, to the incipient appressoria inhibits further development of appressoria (Fig. S5). Cycloheximide treatment appeared to interfere with the nucleolar dynamics during which fungal nucleoli change in accordance with the developmental process. In contrast to the hydrophobic surface, the RFP signals were visible in the nuclei of both conidia and elongated germ tube (hyphae) on the hydrophilic surface (non-inductive surface for appressorium formation) (Fig. S6).

Our observation of the nucleoli in *M. oryzae* revealed that unlike the yeast and animal cells, control of nucleolar activity can be decoupled from nucleolar size in the filamentous fungi under low nutrient availability. This may reflect the mode of proliferation and growth of filamentous fungi in which they should maintain nucleolar activity to enable foraging behavior based on hyphal growth when nutrients are scarce, rather than reduce so much nucleolar activity as to go into a quiescent state. Furthermore, tracing the nucleolus during conidia germination and appressorium formation suggested that the assembly and disassembly of the nucleolus are coordinated with the progression of pathogenic development. With new emerging roles of nucleolus in eukaryotes, our study highlights the gap in existing knowledge on the nucleolus of filamentous fungi and provides the cell biological framework for the genetic dissection of nucleolar functions in fungal development and pathogenesis.

### Fungal strains and culture condition

Wild-type strain KJ201 was obtained from the Center for Fungal Genetic Resources (CFGR), South Korea. The strain expressing *MoNOP1-RFP* was generated by introducing *MoNOP1-RFP* construct (under the native promoter of *MoNOP1*) into the protoplast of KJ201. The selection of transformants was carried out using TB3 agar [0.3% (wt/vol) yeast extract, 0.3% (wt/vol) casamino acid, 1% (wt/vol) glucose, 20% (wt/vol) sucrose and 0.8% (wt/vol) agar powder] supplemented with 400 ppm geneticin. For sporulation, strains were grown in oatmeal agar [OMA; 5% oatmeal (wt/vol) and 2.2% agar (wt/vol)] at 25°C under constant lighting with 1–2 days of aeration. For RNA isolation, 5-day-old hyphae were harvested from the CM broth [0.6% (wt/vol) yeast extract, 0.6% (wt/vol) casamino acid and 1% (wt/vol) sucrose] or MM broth [10% (wt/vol) sucrose, 1% (wt/vol) Ca(NO_3_)_2_·4H_2_O, 0.2% (wt/vol) KH_2_PO_4,_ 0.25% (wt/vol) MgSO_4_·7H_2_O, 0.15% (wt/vol) NaCl] with 120 rpm shacking.

### Western blot analysis

Intactness of MoNOP1-RFP protein was assessed by western blotting. Following 3 days of incubation in liquid media, mycelia were harvested and grinded using liquid nitrogen. Then, mycelial powders in Mg/Np-40 extraction buffer [0.5 M Tris-HCl, 20 mM MgCl2, 2% (vol/vol) beta-mercaptoethanol, 1 mM PMSF, 2% (vol/vol) NP-40, 1× proteinase inhibitor cocktail] were sonicated. After centrifuging sonicated lysates, proteins were separated from supernatant using acetone. Total proteins were separated via 10% SDS-PAGE and transferred to polyvinylidene difluoride (PVDF) membrane using the Mini-PROTEAN System (Bio-Rad, CA, USA). After incubation with antibody, band was detected via FUSION Solo-X (Vilber, Eberhardzell, Germany) and edited using Evolution-Capt software (Vilber, Eberhardzell, Germany). As a primary antibody, anti-RFP antibody (1:2,000; Abcam, Cambridge, UK; ab124754) was used. As a secondary antibody, goat anti-rabbit IgG-H&L (HRP) pre-adsorbed (1:5,000; Abcam, Cambridge, UK; ab7090) was used.

### Fluorescence microscopy

To observe the size of nuclei and nucleoli based on fluorescence signals, sterilized slide glass was coated with CM and MM agar and then inoculated with the spore of the strain expressing MoNOP1-RFP. Nuclei and nucleoli were monitored under the microscope, following the incubation of mycelia in CM and MM agar at 25°C for 3 days and 7 days, respectively. The nuclei were stained using Hoechst 33342 dye (Invitrogen, Waltham, MA, USA) according to the manufacturer’s instructions. For the induction of G1-phase cell cycle arrest, mycelia of the strain expressing MoNOP1-RFP were treated with 200 mM hydroxyurea (HU; Invitrogen, Waltham, MA, USA) at 25°C for 22 hours. The nuclei were stained using Hoechst 33342 dye (Invitrogen, Waltham, MA, USA) according to the manufacturer’s instructions. Microscopy was performed with a Leica DM2500 light microscope, and images were taken with a Leica DFC7000 T digital camera. Excitations were 340 to 380 nm for Hoechst 33342 dye and 515 to 560 nm for RFP. Images were processed using LAS X software. Size of nuclei and nucleoli was measured in diameter using a built-in function in Leica Application Suite V4.0.

### Vegetative growth, reproduction, and appressorium formation

For the measurement of growth, reproduction, and appressorium formation, strains were grown on OMA for 9 days, in three replicates, under constant light condition. Conidia were harvested from 9-day-old colonies using 5 mL of distilled water, counted with hemocytometer under light microscope. To monitor germination and appressorium formation, we counted at least 100 spores at 2 to 8 hours post-incubation (hpi). The concentration of conidial suspension was adjusted to ~5 × 10^4^ spores/mL. A 40 µL of suspension was placed on either hydrophobic surface (coverslips) or hydrophilic surface (slide glass) and then incubated in a humidity chamber at 25°C. The germination and appressorium formation rate were calculated by counting the number of germinating and appressorium-forming conidia at 2, 4, 6, and 8 hpi. All the assays were performed with three replicates in three independent experiments. Slides were examined using a Leica DM2500 light microscope and pictured with a Leica DFC7000 T digital camera using Leica Application Suite V4.0.

### RNA isolation and real-time PCR analysis

RNA was isolated from 5-day-old mycelia collected from CM broth cultures using easy-spin total RNA extraction kit (iNtRON Biotechnology, Seoul, Korea). cDNA was synthesized from 1 µg of total RNA using SuperScript IV First-Strand Synthesis System (Invitrogen, MA, USA). Real-time PCR was conducted in a total reaction volume of 20 µL consisting of 2 µL of cDNA, 1 µM primer mix (forward and reverse), and 10 µL of 2× Power SYBR green PCR master mix (Applied Biosystems, Warrington, UK). Reaction conditions set on the Applied Biosystems 7500 real-time PCR system (Applied Biosystems, Foster City, CA, USA) were 40 cycles of 15 s at 95°C, 30 s at 60°C, and 30 s at 72°C, with three technical replicates. Results of threshold cycles (*C_T_*) were averaged and then normalized as previously described (29). Real-time PCR experiments were conducted with three technical replicates in each of three biological replicates.

### Rice sheath and detached leaf assay

Conidia were collected from 14-day-old oatmeal agar cultures and resuspended to 5 × 10^4^ conidia/mL in deionized water. Susceptible rice seedlings of 4-week-old (Nagdongbyeo) were used for collecting rice sheath segments. Spore suspensions were injected into 4 cm long rice sheaths. Sheath segments were placed in humid plate with a wet paper towel for high humidity and incubated at 25°C. Live cell imaging of infected sheaths was performed after 48 hpi, using Leica DM2500 microscope and Leica DFC7000T digital camera. For detached leaf assay, 40 µL of the suspension (5 × 10^5^ spores/mL) was placed on each detached leaf. Rice leaves were placed in humid plate with a wet paper towel for high humidity and incubated 25°C for 5 days. Images of infected leaves were obtained using a MICROTEK XT3300 scanner at a resolution of 800dpi.

## SUPPLEMENTAL MATERIAL

Fig. S1 mbio.01844-23-s0001.jpg Comparison of KJ201 (wild-type) and the strain expressing MoNOP1-RFP.

Fig. S2 mbio.01844-23-s0002.jpg Size of nuclei and nucleoli in CM and MM with hydroxyurea treatment.

Fig. S3 mbio.01844-23-s0003.jpg Patterns of MoNOP1-RFP signal over time during appressorium development.

Fig. S4 mbio.01844-23-s0004.jpg Effect of transcription and translation inhibitors on conidial germination and appressorium formation.

Fig. S5 mbio.01844-23-s0005.jpg Effect of transcription and translation inhibitors on development of incipient appressorium.

Fig. S6 mbio.01844-23-s0006.jpg Localization of MoNOP1-RFP in spores germinating on hydrophilic surface.

ASM does not own the copyrights to Supplemental Material that may be linked to, or accessed through, an article. The authors have granted ASM a non-exclusive, world-wide license to publish the Supplemental Material files. Please contact the corresponding author directly for reuse.
