# Supplementary figures and images for: Distinct dynamics of the nucleolus in response to nutrient availability and during development in the rice blast fungus
Source: mBio. 2023 Sep 28;14(5):e01844-23. doi: 10.1128/mbio.01844-23 (PMC10653916; doi:10.1128/mbio.01844-23)

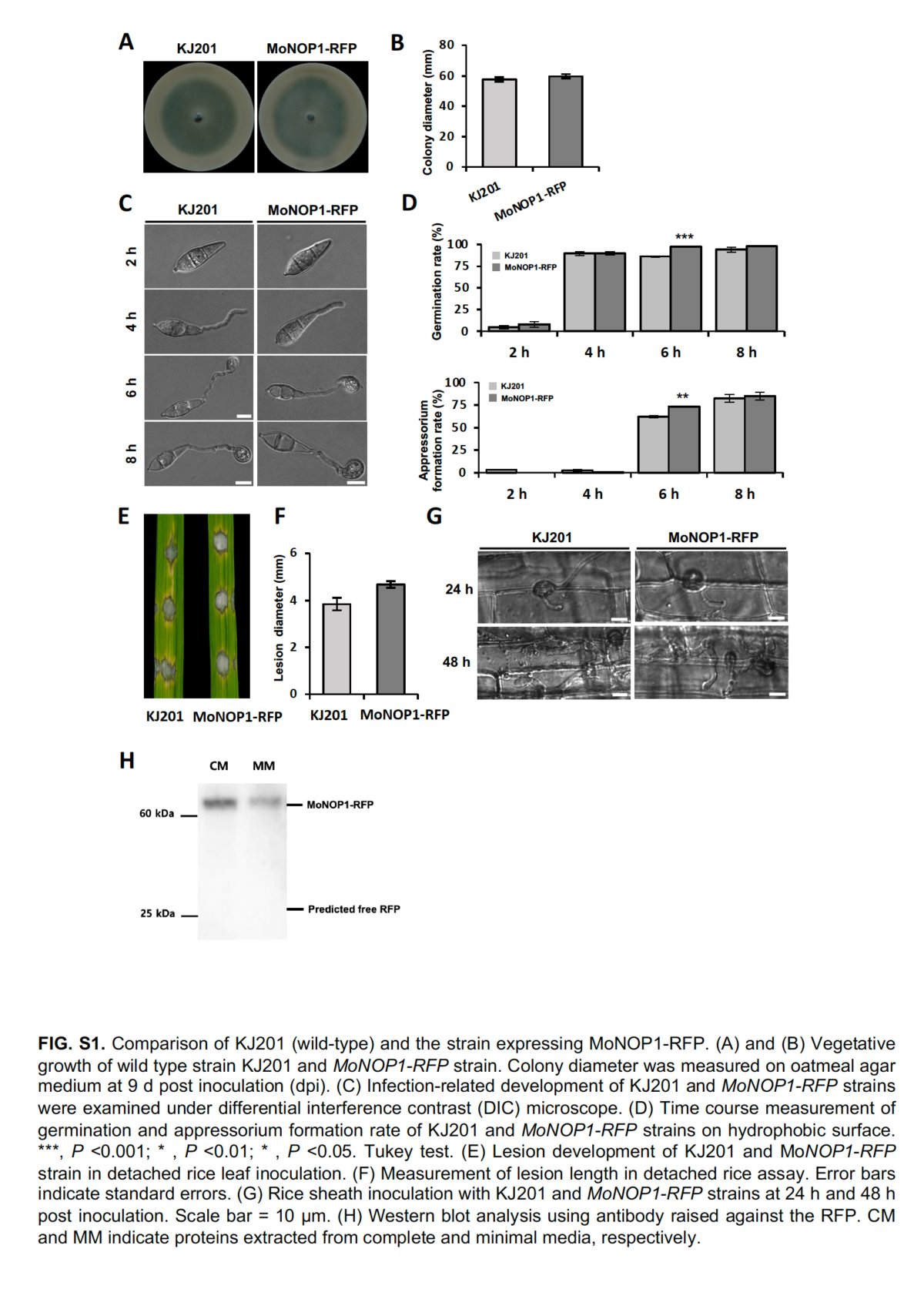

Supplement: Fig. S1 — Comparison of KJ201 (wild-type) and the strain expressing MoNOP1-RFP. [file mbio.01844-23-s0001.jpg]

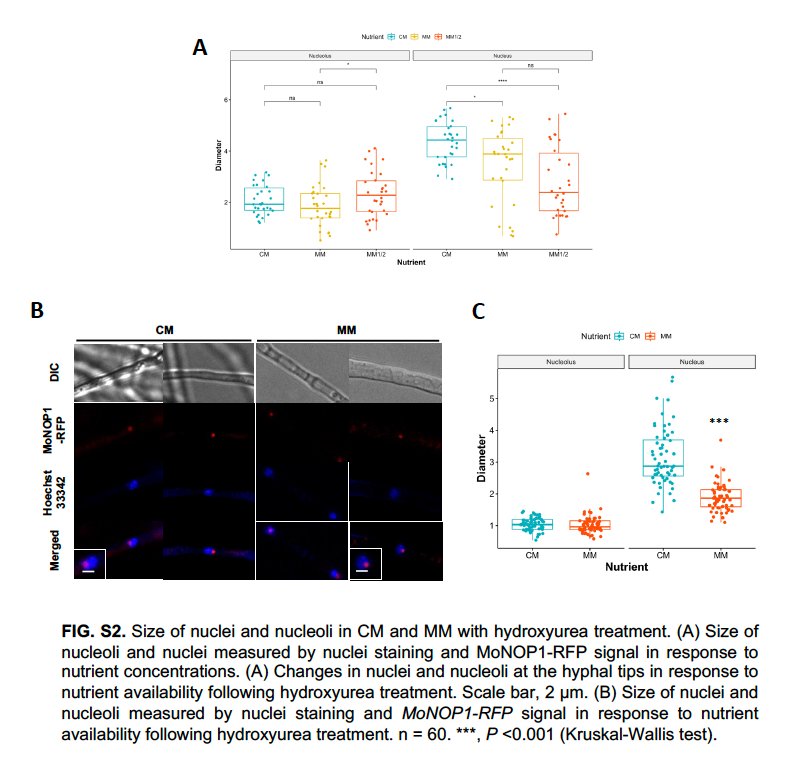

Supplement: Fig. S2 — Size of nuclei and nucleoli in CM and MM with hydroxyurea treatment. [file mbio.01844-23-s0002.jpg]

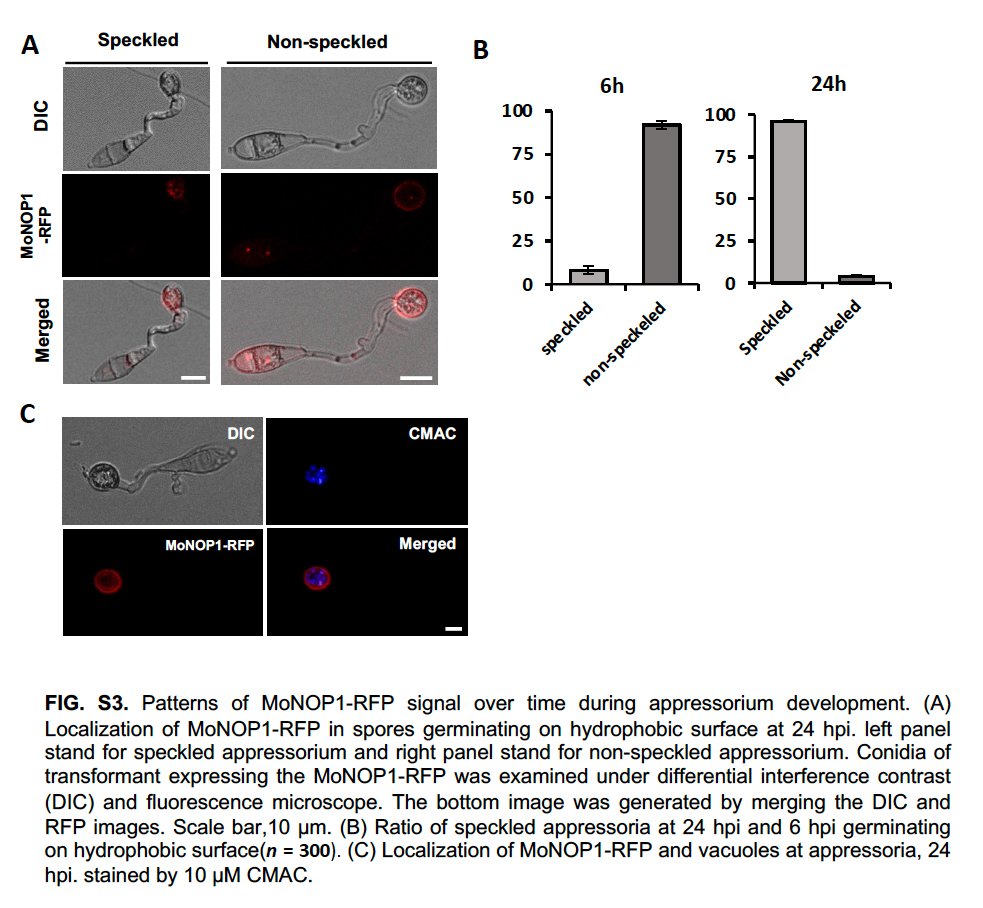

Supplement: Fig. S3 — Patterns of MoNOP1-RFP signal over time during appressorium development. [file mbio.01844-23-s0003.jpg]

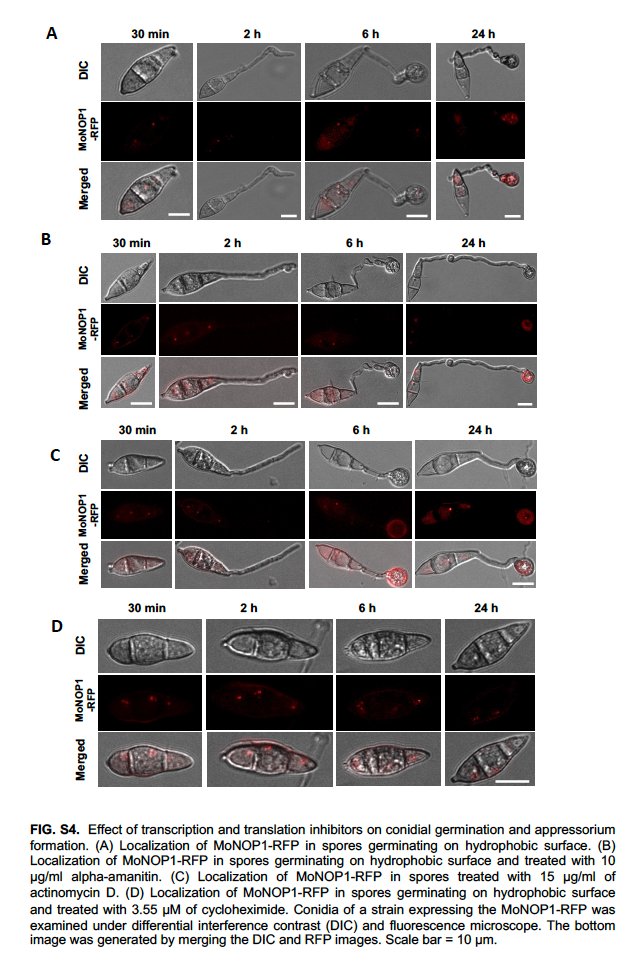

Supplement: Fig. S4 — Effect of transcription and translation inhibitors on conidial germination and appressorium formation. [file mbio.01844-23-s0004.jpg]

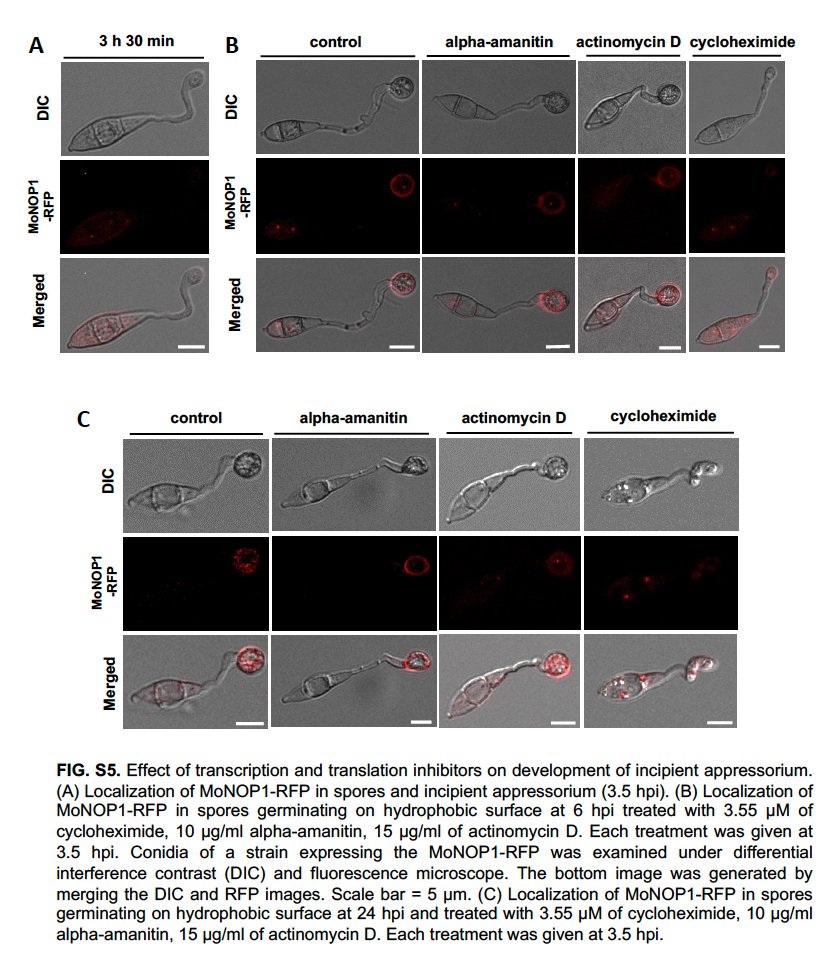

Supplement: Fig. S5 — Effect of transcription and translation inhibitors on development of incipient appressorium. [file mbio.01844-23-s0005.jpg]

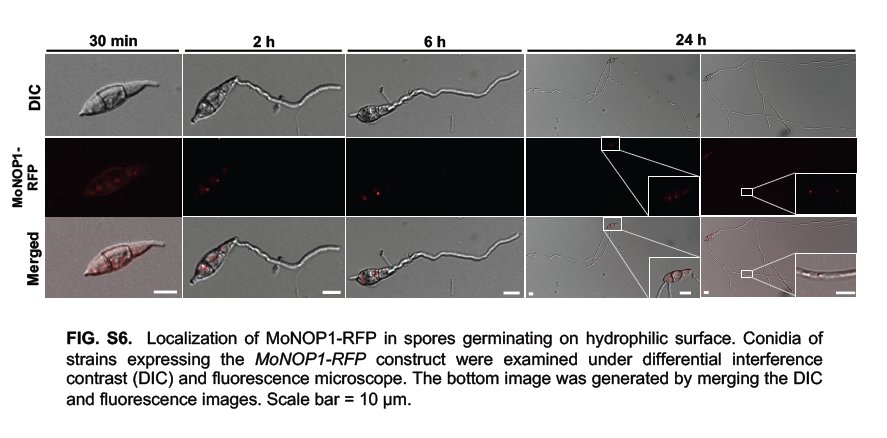

Supplement: Fig. S6 — Localization of MoNOP1-RFP in spores germinating on hydrophilic surface. [file mbio.01844-23-s0006.jpg]
